# Regulation of the Neural Circuitry of Emotion by Compassion Meditation: Effects of Meditative Expertise

**DOI:** 10.1371/journal.pone.0001897

**Published:** 2008-03-26

**Authors:** Antoine Lutz, Julie Brefczynski-Lewis, Tom Johnstone, Richard J. Davidson

**Affiliations:** 1 University of Wisconsin, Madison, Wisconsin, United States of America; 2 West Virginia University, Morgantown, West Virginia, United States of America; 3 University of Reading, Reading, United Kingdom; James Cook University, Australia

## Abstract

Recent brain imaging studies using functional magnetic resonance imaging (fMRI) have implicated insula and anterior cingulate cortices in the empathic response to another's pain. However, virtually nothing is known about the impact of the voluntary generation of compassion on this network. To investigate these questions we assessed brain activity using fMRI while novice and expert meditation practitioners generated a loving-kindness-compassion meditation state. To probe affective reactivity, we presented emotional and neutral sounds during the meditation and comparison periods. Our main hypothesis was that the concern for others cultivated during this form of meditation enhances affective processing, in particular in response to sounds of distress, and that this response to emotional sounds is modulated by the degree of meditation training. The presentation of the emotional sounds was associated with increased pupil diameter and activation of limbic regions (insula and cingulate cortices) during meditation (versus rest). During meditation, activation in insula was greater during presentation of negative sounds than positive or neutral sounds in expert than it was in novice meditators. The strength of activation in insula was also associated with self-reported intensity of the meditation for both groups. These results support the role of the limbic circuitry in emotion sharing. The comparison between meditation vs. rest states between experts and novices also showed increased activation in amygdala, right temporo-parietal junction (TPJ), and right posterior superior temporal sulcus (pSTS) in response to all sounds, suggesting, greater detection of the emotional sounds, and enhanced mentation in response to emotional human vocalizations for experts than novices during meditation. Together these data indicate that the mental expertise to cultivate positive emotion alters the activation of circuitries previously linked to empathy and theory of mind in response to emotional stimuli.

## Introduction

Many contemplative traditions speak of loving-kindness as the wish of happiness for others, and of compassion as the wish to relieve others' suffering. In many traditions, these qualities are cultivated through specific meditation practices designed to prime behaviors compatible with these wishes in response to actual interpersonal encounters. Despite the potential social and clinical importance of these affective processes, the possibility that they can be trained in a manner comparable to attentional [1] or sensory-motor skills [2] has not yet been investigated with neuroimaging techniques, even though recent electrophysiological data support this hypothesis [3].

To cultivate these affective qualities practitioners in a number of traditions have developed meditative practices, which are thought to be essential to counteract self-centered tendencies [4]. Techniques include concentration exercises that train attention, behavioral training such as the practice of generosity, cognitive strategies including reflection on the fleeting nature of the self and empathic strategies such as shifting perspectives from self-oriented to other-oriented, or the visualization of the suffering of others [5]. Traditionally such mental training comprises years of scholastic study and meditative practice. The long-term goal of meditators undergoing such training is to weaken egocentric traits so that altruistic behaviors might arise more frequently and spontaneously. The purpose of this study is to examine the brain circuitry engaged by the generation of a state of compassion (short for “compassion and loving-kindness meditation state”) in long-term Buddhist meditators and novice meditators.

Here, “expert” meditators have more than 10,000 hours of practice in Buddhist meditation and are perceived in their communities as embodying qualities of compassion (see Methods). Experts were compared with age-and gender-matched “novices” who were interested in learning to meditate, but had no prior experience except in the week prior to the scanning session, in which they were given meditation instructions for the same practice performed by the experts. The meditative practice studied here involves the generation of a state in which an “unconditional feeling of loving-kindness and compassion pervades the whole mind as a way of being, with no other consideration, or discursive thoughts” (for details see Meditation Instruction). According to the tradition, as a result of this practice, feelings and actions for the benefit of others arise more readily when relevant situations arise. Our main hypothesis was thus that the concern for others cultivated during this meditation would enhance the affective responses to emotional human vocalizations, in particular to negative ones, and that this affective response would be modulated by the degree of meditation training. Here we broadly refer to empathy as the capacity to understand and share another person's experience. Recent fMRI or PET studies have demonstrated that observing or imaging another person's emotional state activates parts of the neuronal network involved in processing that same state in oneself, whether it is disgust, pain, or social emotion [6], [7] (for reviews see [8], [9]). These data are consistent with perception-action models of empathy [10] in which observing and imagining another person in a particular state is thought to activate a similar state in the observer.

Brain function was interrogated using a block and event-related paradigm during periods of mental practice alone, and in response to emotional human vocalizations (positive, neutral, or negative sounds from a normalized database, [11]). The block and event-related effects were modeled as independent factors in the analysis. To test our main hypothesis, we focus here only on the event-related data that allow the study of the modulation of responses to emotional stimuli by this voluntarily induced state. The voxel-wise analysis of the emotional sounds (event-related design) was performed using a 2×2×3 factorial design with the first factor representing “Group” (15 experts vs. 15 novices), the second factor “State” (compassion vs. rest), and the third factor “Valence” (negative, neutral or positive emotional sounds). We predicted that participants would feel more moved by the emotional sounds during compassion meditation than when at rest. Thus, the brain regions underlying emotions and feelings (insula, anterior cingulate cortex (ACC), and possibly somatosensory areas, for review see [12], [13], [14]) would be more activated in response to emotional sounds during compassion meditation than during the resting state. As this meditation is said to enhance loving-kindness when the joy of others is perceived or compassion when the suffering of others is perceived, this effect was predicted to be stronger for the negative sounds (sounds of a distressed woman) and positive sounds (a baby laughing) than for neutral sounds (background noise in a restaurant). As this state is practiced to foster altruistic behaviors, the predicted three-way interaction (Group by State by Valence) should be driven by a stronger empathic response to negative than the positive sounds during meditation than rest and a modulation of this effect by expertise.

In this study we did not include a behavioral task because practitioners reported that a task would disrupt their ongoing meditation. But verbal self-reported intensities of the meditation were collected after each block allowing us to identify good vs. poor blocks of meditation (see protocol). To further confirm our general prediction, we examined the interaction between the verbally reported quality of meditation (good vs. poor) and Group as factors. We predicted that insula and ACC would be more activated in response to emotional sounds during good vs. poor block of compassion, as verbally reported. Finally, we measured pupil diameter to obtain an independent index of autonomic arousal [15] (eyes open and loosely fixated on a fixation point in both rest and meditation blocks) to determine if there were group differences in autonomic arousal during the task. To eliminate any possible group differences in autonomic arousal from influencing MR signal changes, we regressed out the effect of pupil dilation from BOLD responses in the empathic circuitry to remove the contribution of variations in emotional arousal from empathic responses.

## Results

**Figure 1:**
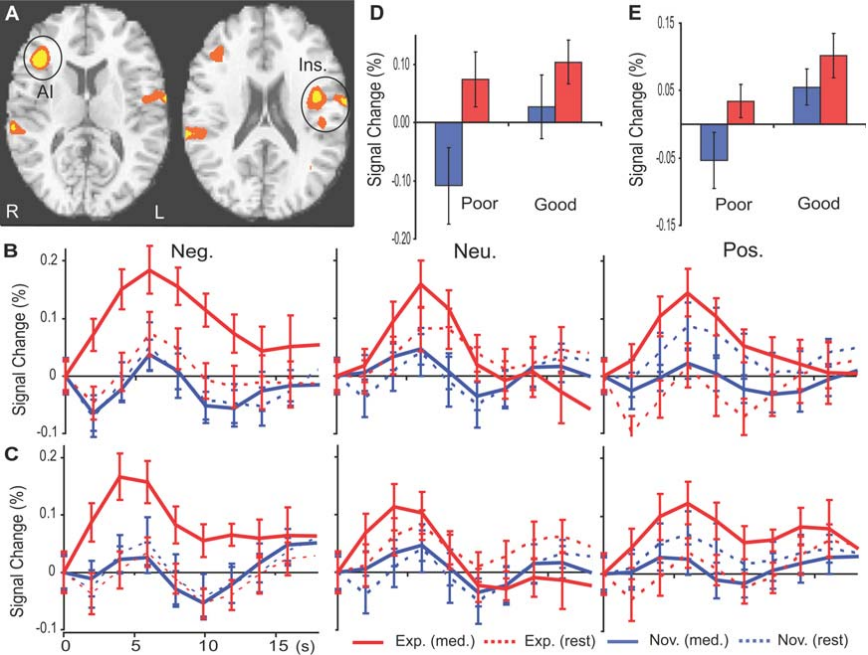
State by Group by valence Interaction: A. (AI) and (Ins.) stand for anterior insula and insula, respectively (z = 12 and z = 19, 15 experts and 15 novices, color codes: orange, p<5.10ˆ-2, yellow, p<2.10ˆ-2). B, C. Impulse response from rest to compassion in response to emotional sounds in AI (B) and Ins. (C). D–E. Responses in AI (D) and Ins. (E) during poor and good blocks of compassion, as verbally reported, for 12 experts (red) and 10 novices (blue).

**Figure 2:**
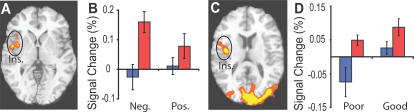
Meditation modulates right insula response to emotional sounds: A. Voxel-wise analysis of the Group by State by Valence (negative versus positive sounds) interaction in insula (Ins.) (z = 2, corrected, colors code: orange, p<5.10ˆ-2, yellow, p<2.10ˆ-2, 15 experts (red) and 15 novices (blue)). B. Average response in Ins. from rest to compassion for experts (red) and novices (blue) for negative and positive sounds. C–D. Voxel-wise analysis of BOLD response to emotional sounds during during poor vs. good blocks of compassion, as verbally reported. C. Main effect for verbal report in insula (Ins.) (z = 13, corrected, colors: orange, p<10ˆ-3, yellow, p<5.10ˆ-4, 12 experts and 10 novices). D. Average response in (Ins.) for experts (red) and novices (blue).

As predicted there was a Group-by-State-by-Valence interaction in several regions critical for empathy (insula cortex, somatosensory cortex (SII), Fig. 1.A, Table 1). The interaction was a function of experts showing a larger increase than the novices during meditation vs. rest in response to emotional (positive and negative) vocalizations vs. neutral vocalizations (Figs. 1.B–C, Table 1). The activation in insula cortex during compassion was a function of the intensity of the meditation as verbally reported, which was stronger during the good vs. the poor blocks of meditation across the two groups (Figs. 1. D–E, Table 1). Since there was no difference between states in response to the neutral sounds in the clusters from figure 1 (table 1), following our prediction we ran a follow-up 2*2*2 ANOVA using only negative and positive sounds in a voxel-wise analysis. There was only one cluster showing a Group by State by Valence interaction, which was located in the right insula (3667 voxels, corrected p<0.05, Fig. 2.A). The effect was produced by a stronger activity in the responses to negative vs. positive sounds during meditation vs. rest for the experts compared to the novices (Fig. 2.B, t = 2.1, df  = 28, P<0.05, paired t test). The activity in this cluster was also stronger during the good vs. poor blocks of meditation (main effect for verbal report, F(1,20) = 6.8, P<0.05, ANOVA). The voxel-wise 2*2 repeated ANOVA analysis with group and verbal report (poor vs. good) as factors confirmed these findings. There was a main effect for good vs poor blocks in the right insula (Figs. 2.C–D) and ACC (Table 2). Together these results support our main hypothesis that the brain regions underlying emotions and feelings are modulated in response to emotional sounds as a function of the state of compassion, the valence of the emotional sounds and the degree of expertise.

**Table 1:** Group by State by Valence interaction.

ROIs, Group by State by Valence interaction	L/R	Vol mm^3^	Talairach coord. x y z	Gr. by St. by val. Inter. F-value	Neu (Med. vs Rest), t-value	(Pos+Neg)/2-Neu, t-value	Main effect verbal report, F-value	Interaction verbal report by Group, F-value
Ant. Insula/Inf. Frontal gyrus	R	3822	38 20 12/39 24 11	4.6	X (p>0.4)	2.3 (E>N)	6.6 (G>P)	X
Insula/post-/precentral gyrus	L	5005	−40 −1 17/−60 −6 18	4.3	X (p>0.4)	2.2 (E>N)	5.3 (G>P)	X
Sup.temporal gyrus/Inf. Parietal lobule (SII)/ post-central gyrus/ Insula	R	4802	60 −29 14/52 −26 27/54 −22 32/49 −30 19	4.6	2.3 (Med.>Rest)	2.2 (E>N)	X	X
Precuneus/Sup. Parietal Lobule	L	4102	−20 −54 39/−19 −62 53	4.1	X (p>0.4)	2.2 (E>N)	6.2 (G>P)	X

Clusters extracted from the ROIs showing Group by State by Valence interaction (p<0. 05 corrected, repeated ANOVA). T-test comparing responses during meditation minus resting states for experts (E) vs. novices (N) during neutral sounds (Neu.) and during either positive and negative sounds minus neutral sounds ((Pos+Neg)/2-Neu) were run within in each of these ROIs (p<0.05). A 2^*^2 repeated ANOVA with verbal report (poor (P) vs. good (G)) and groups (experts vs. novices) was run in these ROIs on the response to positive and negative sounds during meditation blocks (12 experts and 10 novices). X indicates non-significant (p>0.05).

**Table 2 pone-0001897-t002:** Activation during poor vs. good blocks of meditation, as verbally reported.

ROIs, main effect for verbal report	L/R	Vol mm^3^	Talairach coord. x y z	F value (best>worst blocks)
Occipital gyrus/Cuneus/Lingual gyrus	L/R	18930	−30 −81 21/ 7 −80 18	41.5**
ACC/ Middle frontal gyrus	L/R	8790	−3 18 37/33 32 36	19.3**
Paracentral lobule/post. medial frontal gyrus	L/R	6474	−6 −37 58/−1 −8 55	16.4*
Hippocampus/Parahippocampus	L/R	3549	−32 −14 −11	17.4**
Insula/IFG	R	2490	37 −7 12	19.8**
**ROIs, interaction verbal report and Group**	**L/R**	**Vol mm^3^**	**Talairach coord. x y z**	**F value Novices (best –worst)>Experts (best-worst)**
Cuneus/Lingual gyrus	L/R	20164	−7 −92 9/−10 −84 2	31.8**
Brain stem		7328	−2 −18 −25	21.2**
thalamus	L/R	5416	14 −18 15	11.7*
Cerebellum	L/R	3629	14 −15 −62	12.7*

ROIs from a voxel-wise repeated ANOVA with best vs. worst blocks of meditation as within subjects factor and experts vs. novices as between subjects factor (10 novices and 12 experts) (p<0.05, corrected).

* indicates p<0.005,

** p<0.0005, X indicates non-significant.

In addition, we explored the other effects from our main 3*2*2 factorial design. There was no main effect of group. The main effect for state showed stronger activation during meditation than rest in limbic regions (AI, ACC) and in a circuitry previously linked with “mentation” about the mental states of others (temporal lobes, pSTS, TPJ, medial prefrontal cortex (mPFC) and the posterior cingulate cortex (PCC)/ precuneus (Prc.)) (Table 3). The pattern exhibited stronger activity in the right hemisphere than in the left hemisphere (Table 4).

**Table 3 pone-0001897-t003:** Main effect for State.

ROIs, main effect for State	L/R	Vol mm^3^	Talairach coord. x y z	State effect F-value	Novices t-value (Med.>Rest)	Experts t-value (Med.>Rest)
Precuneus	L/R	17835	−5 −55 52	32.7**	X	5.8**
Supramarginal gyrus (TPJ)	R	715	52 −43 37	38.2**	X	6.8**
Inf. Parietal lobule (including SII)	L/R	7453	46 −40 47	43.7**	2.8	6.1**
Ant. Insula	L/R	1963	37 15 1	27.2**	X	4.8**
Sup. Temporal sulcus (post)	L/R	6267	54 −38 14	35.5**	X	5.7**
Sup. Temporal gyrus (ant.)	L/R	3416	54 5 −7	28.2**	X	5.2**
Sup. Parietal lobule	R	948	34 −60 46	28.6**	X	6.1**
Post. cingulate gyrus	L/R	9185	6 −40 40	35.7**	2.4	5.5**
Mid. temporal gyrus (post)	R	6280	58 −47 −3	38.6**	X	6.4**
Parahippocampal gyrus	L/R	357	12 −39 4	22.3**	X	5.3**
Fusiform gyrus	L/R	1629	48 −38 −17	21.8**	X	4.93**
Cerebellum	L/R	2471	16 −48 −12	24.8**	X	6.1**
Brain stem	L/R	591	3 −22 −6	26.4**	X	X
Ant. cingulate gyrus/medial frontal gyrus	R	4372	5 24 37/9 6 42	28.06**	4.5**	4.1*
Mid. frontal gyrus	R	1027	22 9 58	19.1**	3.0	3.4*

Clusters extracted from the ROIs showing State effect (p<0. 05 corrected, repeated ANOVA). Paired t-tests comparing responses during meditation and resting states were run within each group in each of these ROIs (p<0.05).

* indicates p<0.005,

** p<0.0005, X indicates non-significant.

**Table 4 pone-0001897-t004:** Laterality effect.

ROIs, main effect for State	Vol mm^3^	Talairach coord. x y z	Side effect	State by Side effect	State by Side by Group effect
Pars Operculum (SII)	5491	46 −31 32	X	8.7 (R>L)	X
Supramarginal gyrus (TPJ)	1623	52 −44 35	X	13.9* (R>L)	5.7
Inf. Parietal Lobule	6919	47 −46 44/46 −41 29	X	10.8* (R>L)	X
Sup. Temporal sulcus	4434	48 −35 15	X	13.4* (R>L)	X
Mid. temporal gyrus	6277	52 −48 −5	X	22.2**	X
Fusiform gyrus	3446	48 −38 −17	X	5.4 (R>L)	X
Parahippocampal gyrus	3009	12 −38 4	X	6.8 (R>L)	X
Ant. cingulate gyrus/medial frontal gyrus	5040	4 35 27/4 21 44	X	11.1* (R>L)	6.3

Laterality effects were tested in the clusters showing state effect (meditation versus resting states) (Table 1) or Group by State effect (Table 2). Repeated ANOVA with Laterality, State and Group as factors were run on the average activity in these ROIs and in symmetrical ROIs in the other hemisphere. p<0.05).

* indicates p<0.005,

** p<0.0005, X, L, R indicate non-significant, left and right respectively.

**Figure 3:**
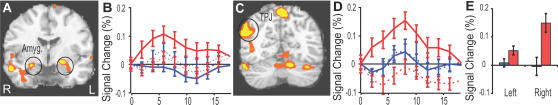
State by Group Interaction: A. (Amyg.) stands for amygdala (y = −5, color codes: orange, p<2.10ˆ-3, yellow, p<5.10ˆ-4). B. Impulse response in (Amyg.) for 15 experts (red) and for 15 novices (blue) during rest (dashed line) and compassion (full line). C–D. Same as A–B in TPJ; y = −61. E. Side by state effect and side by state by group effect in TPJ on the average impulse response between meditation and rest; experts are in red, novices in blue.

**Figure 4:**
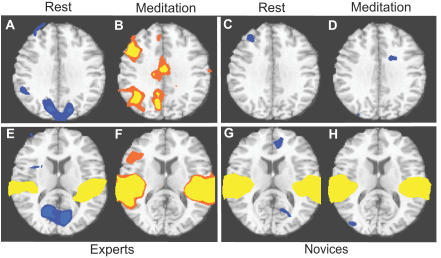
Directionality of the brain activation. Areas showing a negative ( dark blue, p<0.01, blue, p<0.005) or positive (orange, p<0.01, yellow, p<0.005) impulse response on average across 10 seconds in responses to all emotional sounds for the 15 novices and 15 experts at z = 31 compared to baseline (figs. A–D) and z = 13 (figs. E–H) (voxel-by-voxel paired t test compared to 0, corrected at p<0.01).

There were no Group-by-Valence or State-by-Valence interactions. Voxelwise analysis of group-by-state interactions showed experts to have had considerably stronger activation in components of the posterior part of this network (right TPJ, right pSTS, Prc./PCC) (Figs. 3.C–D), in the right inferior frontal gyrus (IFG), bilateral amygdalae (Figs. 3.A–B) and in two motor regions (pre-central gyri and post. medial frontal cortex, BA6) (Table 5). The magnitude of the group-by-state interaction was driven by the BOLD response of experts, who showed a negative average impulse response to the sounds at rest (Figs. 4.A and 4.E) but a positive response in these regions during meditation (Figs. 4.B and 4.F) in right TPJ, right IFG, Prc./PCC and mPFC. Novices and experts showed similar positive activation in the auditory cortex during both rest and meditation, indicating, as expected, sensory correlates of the auditory sounds (Figs. 4.E–H). These group differences were also highlighted in patterns of asymmetric BOLD response in the TPJ where experts showed a strong right-sided activation bias while novices showed virtually no activation difference to the emotional sounds in this region during meditation vs. rest (Fig. 3.E, Table 6).

**Table 5:** Group by State interaction.

ROIs, Group by State interaction	L/R	Vol mm^3^	Talairach coord. x y z	State effect F-value	Group effect F-value	State by Group Interact F-value	Novices t-value (Med.>Rest)	Experts t-value (Med.>Rest)
Angular gyrus (TPJ)	R	1128	51 −59 35	15.7**	X	18.5**	X	4.6**
Supramarginal gyrus (TPJ)	R	828	44 −52 27	19.1**	X	19.8**	X	4.8**
Inferior Parietal Lobule (post.)	R	392	45 −58 35	12.8*	X	12.46*	X	3.9*
Sup. Temporal gyrus (ant.)	R/L	2889	55 −12 0	22.5**	X	18.6**	X	5.0**
Sup. Temporal sulcus (post.)	R/L	4917	45 −45 10	21.2**	X	23.9**	X	5.6**
precuneus		6582	−2 57 54	23.8**	X	26.7**	X	6.4**
Mid. Temporal gyrus (post.)	R/L	3854	62 −38 5	17.1**	4.5	26.75**	X	5.26**
Mid. Temporal gyrus (ant.)	R/L	1933	55 −8 −16	12.6*	X	24.9**	X	5.3**
Post. cingulate gyrus	L/R	1871	6 −48 35	32 **	X	24.6**	X	6.2**
Medial frontal gyrus/paracentral lobule	L/R	5317	−3 −24 67	6.4	X	19.5**	X	4.1*
Post- and pre-central gyri	L	7121	−32 −27 59/ 36 −16 49	6.2	X	20.3**	X	4.2*
Cerebellum	L/R	13528	−20 −69 −17	8.3	X	20.2**	X	4.7**
Lentiform nuclei and Globus Pallidus	L/R	526	−16 −9 −7	15.5**	X	21.6**	X	4.97**
Amygdala	L/R	484	22 −6 −10	4.5	X	19.4**	X	4.12*
Thalamus	R	401	8 −24 10	7.1	X	16.4**	X	6.3**
IFG	R	1266	39 27 −13	8.3	X	16.5**	X	4.5*

Clusters extracted from the ROIS showing State effect (p<0. 05 corrected, repeated ANOVA). Paired t-tests comparing responses during meditation and resting states were run within each group in each of these ROIs (p<0.05).

* indicates p<0.005,

** p<0.0005, X indicates non-significant.

**Table 6:** Laterality effect.

ROIs, from State by Group interaction	Vol mm^3^	Talairach coord. x y z	Side effect F-value	State by Side effect F-value	State by Side by Group effect F-value
Sup. Temporal sulcus (post.)	4917	55 −12 0	X	14.9* (R>L)	X
Mid. temporal gyrus (post.)	3854	62 −38 5	X	15* (R>L)	X
Supramarginal gyrus (TPJ)	828	44 −52 27	X	8.5* (R>L)	15.9**(R>L)
Angular Gyrus (TPJ)	1128	51 −59 35	X	7.2 (R>L)	10.7*(R>L)
Cerebellum	13528	−20 −69 −17	X	11.2* (R>L)	X
IPL	392	45 −58 35	X	4.5 (R>L)	6.2 (R>L)
thalamus	401	8 −24 10	X	X	21** (R>L)

Laterality effects were tested in the clusters showing group by state effect (Table 3). Repeated ANOVA with laterality, state and group as factors were run on the average activity in these ROIs and in symmetrical ROIs in the other hemisphere. p<0.05).

* indicates p<0.005,

** p<0.0005, X, L, R indicate non-significant, left and right respectively.

Finally, pupil diameter increased in response to all sounds in meditation vs. rest (9 controls and 7 experts participants, main effect for state, ANOVA, F(1,15) = 5.2, p<0.05) and was stronger for experts than novices (group by state interaction, ANOVA, F(1,15) = 5.2, p<0.05). The pupil diameter increase during meditation vs. rest positively correlated with the larger increase in anterior insula (AI) in responses to all sounds in meditation vs. rest (r = 0.54, p<0.05). In this cluster, there was a State-by-Group interaction, as well as a State effect, (stronger for experts than novices, ANOVA, F(1,28) = 11.3, p<0.005 ) that was preserved even when the variation in the pupil signal was covariated out from the BOLD signal (ANCOVA, F(1,27) = 20.2, p<0.0005 for State effect and F(1,27) = 5.1, p<0.05 for State–by-Group interaction).

## Discussion

Prior neuroimaging studies of empathy have shown that by observing another's emotional state, part of the neural circuitry underlying the same state becomes active in oneself, whether it is disgust, pain or social emotions (see [6]–[9]. Such findings are consistent with the perception-action model of empathy [10]. Recently, researchers have begun to investigate whether these empathy processes can be modulated by the implicit context of the empathic experience [8], [16]. We extended this contextual approach by showing that regions previously associated with empathic processes were modulated by voluntary regulation of one's emotional responses via the generation of compassion.

All participants exhibited stronger neural responses to all emotional sounds in the AI and ACC during compassion meditation than when at rest (Table 3), and experts exhibited stronger responses than novices to negative than to positive emotional sounds in somatosensory regions (SII, post-central gyrus) during compassion meditation than when at rest (see Fig. 1.A–B, Table 1). Those regions in which stronger activity was measured are also known to participate in affect and feelings [12], [13], [14]. Furthermore, the amplitude of the activity in several of these regions, in particular the insula cortex, was associated: with the degree to which participants perceived that they had successfully entered into the meditative state (Figs. 1.D–E and 2.C–D, Table 2); with expertise of compassion meditation (Figs. 1.A–C, 2.A–B, Tables 1 and 5); and with the relevancy of the emotional sounds during the compassion meditation (stronger response to the voice of a distressed person than that of a laughing baby, or than to background noise from a crowd, 1.A–C, 2.A–B, Table 1). The peaks of activation in the IA (x = 37, y = 15, z = 1, Table 3) and ACC (one at (x = 9, y = 6, z = 42), and one at (x = 5, y = 24, z = 37), Table 3)) found in the main effect of state (compassion vs. rest) overlap with regions previously found to be activated during empathy for others' suffering (x = 39, y = 12, z = 3) for IA and ACC (x = −9, y = 6, z = 42) and (x = 0, y = 24, z = 33) from [6]. A similar interaction effect in the somatosensory cortex was found in this brain region, reflecting greater activation when adopting the first-person vs. third-person perspectives, and even more during an emotional vs neutral context [7]. These findings suggest that cultivating the intent to be compassionate and kind can enhance empathic responses to social stimuli. The functional group difference found in insula is consistent with larger cortical thickness in this region among meditators than among controls, in a group of meditators trained in a tradition that usually contains a compassion meditation component [17]. The group difference in BOLD signal is consistent with the group difference in amplitude of gamma-band (25–50 Hz) oscillations in EEG data recorded from the same group of long-term meditators during the same meditation [3].

We found greater activation in a circuit commonly recruited during the reading of others' mental states (TPJ, pSTS, mPFC, PCC/Prc., Figs 3–4. , Tables 3 and 5) in response to sounds during compassion than when at rest [9], [18], [19]. This pattern was strongly modulated by expertise in particular in the PCC/Prc and right pSTS/ TPJ (Figs. 3.C–D, Table 5). Many of these regions were lateralized to the right (Table 4) more strongly for experts than for novices, particularly in the right TPJ (Fig. 2.E, Table 4). The right lateralization of pSTS is in accordance with previous work on social cognition [9], [18]. Of particular interest to our study, the link between expertise in compassion and the activation in the right pSTS is consistent with the finding that pSTS activation predicts self-reported altruism [20]. The activation peak in pSTS in this study was part of the cluster illustrated in Fig. 3.C (x = 46, y = −64, z = 23) and of the cluster from the main effect of state (x = 41, y = −48, z = 45, Table 3). Our finding of greater activation in the right pSTS/ TPJ among experts suggests that the meditative practice of compassion may enhance emotion sharing, as well as perspective taking.

In addition to the right pSTS/TPJ, the scope of the brain circuitry which showed an interaction between expertise and meditation also encompassed the right IFG (Table 5). The TPJ and IFG together compose a circuitry classically viewed as an attentional system specialized to detect behaviorally relevant stimuli, in particular when the stimuli are salient or unexpected [21]. A similar increase of activation in the amygdalae, linked to appraisal of emotional stimuli, (Figs. 3.A–B, Table 5) further supports this view. The greater increase in activation of this circuitry in experts than in novices suggests that experts might be more primed to detect salient events, such as the suffering of others, during this voluntarily induced state. Even if attention might have influenced the processing of emotional stimuli and thus have increased emotional arousal, the fact that the activation in the insula was still present when we regressed out changes in pupil diameter induced by the sounds supports the role of insula not only in emotional arousal, but also in empathic processes.

Most of the areas included in the “mentation network” also overlap with the proposed “default mode” or “resting state” networks (typically mPFC, rostral ACC, PCC, Prc and posterior lateral cortices, for review [19], [22]). A wide range of tasks have been found to produce a relative decrease in BOLD signal in this network in comparison to a passive resting state, implying that this network is also active during the resting state. Given recent interest in this network, it is worth noting the experts' ability to generate states that can selectively produce BOLD deactivation (rest, Figs. 4.A, 4.E) and activation (meditation, Figs. 4.B and 4.F) in precuneus and TPJ in response to sounds, suggesting that these regions were more activate during rest than meditation prior to the presentation of the sounds. Future study investigating in more detail the phenomenology of these states might shed new light on the functionality of this circuitry.

Because novices and experts differ in many respects other than simply the extent of meditative training (such as culture of origin and first language), longitudinal research that follows individuals over time in response to compassion training will be needed to further substantiate our findings. It will also be essential to assess the impact of such emotional training on behavioral tasks involving altruism, and, more generally, emotional reactivity and regulation. The long-term question is to evaluate whether repeated practice in such techniques could result in enduring changes in affective and social style [23]. The fact that large and systematic changes in brain function were observed in response to auditory emotional stimuli presented during the meditative practice of compassion, and the fact that robust differences were observed between experts and novices, suggests that the next steps to evaluate the behavioral impact of this training and to longitudinally assess its effects are warranted.

## Methods

### Participants

Participants included 16 long-term Buddhist meditators, whom we classified as experts (mean = 45.0 years, SD, 12.7 years, ages 29 to 64 years for the 15 experts used in these analyses), and 16 healthy volunteers (ages 36 to 56 years, mean = 47.1 years, SD 8.8 for the 15 novices used in these analyses who did not differ in age (t test, p = 0.55). Two participants were not included in the analysis due to excessive motion (see Data Analysis). All participants were right-handed, except for one ambidextrous expert, as assessed by Edinburgh Handedness Inventory [24], and all but 4 were male (2 experts and 2 age-matched novices). Buddhist meditators recognized as experts (9 of Asian origin, 7 of European origin) were contacted by Dr. Ricard, an interpreter for the Dalai Lama who is a Western Buddhist monk with scientific training and 35 years of meditative training in Nepal. Experts had previously completed from 10,000 to 50,000 hours of meditative training in a variety of practices, including compassion meditation, in similar Tibetan traditions (Nyingmapa and Kagyupa). The length of their training was estimated based on their daily practice and time spent in meditative retreats. Ten hours of meditation per day of retreat was estimated as an average. Control participants were recruited via advertisements in local newspapers and consisted of members of the UW Madison community. The advertisement specifically recruited participants who had an interest in meditation, but who had had no prior meditative training. One week before the actual fMRI scan session, novices were given written instructions on how to perform the meditative practices, written by Dr. Ricard, following which they practiced this compassion meditation and two other meditations for one hour a day for a week (20 minutes per meditation). Written informed consent was obtained prior to scanning, in accordance with procedures and protocols approved by the UW-Madison Institutional Review Board. A proficient Tibetan speaking translator gave detailed procedural instructions and read the consent form to non-English speaking participants.

### Meditative instruction

The state of loving-kindness and compassion is described as an “unconditional readiness and availability to help living beings”. This practice does not require concentration on particular objects, memories or images, although in other meditations that are also part of their long-term training, meditators focus on particular persons or groups of beings. Because “benevolence and compassion pervades the mind as a way of being”, this state is called “pure compassion” or “non-referential compassion” (dmigs med snying rje in Tibetan). As described in Dr. Ricard's instructions for novices: “During the training session, the subject will think about someone he cares about, such as his parents, sibling or beloved, and will let his mind be invaded by a feeling of altruistic love (wishing well-being) or of compassion (wishing freedom from suffering) toward these persons. After some training the subject will generate such feeling toward all beings and without thinking specifically about someone. While in the scanner, the subject will try to generate this state of loving kindness and compassion.” The Resting state (Tib. “sem lung ma bstan”–literally: neutral (lung ma ten) mind (sem)) was a non-meditative state without specific cognitive content and with a lack of awareness or clarity of the mind. Novice's Instructions were the following: “Neutral here means that your emotional state is neither pleasant nor unpleasant and that you remain relaxed. Try to be in the most ordinary state without being engaged in an active mental state.” Novices' ability to follow the instruction was assessed orally prior to the data collection.

### Protocol

Before the MRI scanning session, participants had a simulation session during which they viewed an abbreviated version of the experimental paradigm while lying in a mock MRI scanner (including head coil and digitized scanner sounds). This simulation session served to acclimate participants to the fMRI environment. We used a block design, alternating ∼3 min of the state of meditation (4 cycles) with ∼1.6 min of a resting, neural state (5 cycles), twice on separate days. There was an average time per session of 643 seconds of meditation and 550 seconds of neutral state (264 seconds and 190 seconds respectively for expert participant 2). A total of 25 2-second auditory sounds from the International Affective Digitized Sounds (IADS) [11] for each valence (positive, neutral and negative) were randomly presented across these two sessions. These sounds were presented every 6–10 seconds after the first 40 seconds of the meditative blocks and after 15 seconds of the resting blocks. To have a comparison condition for the statistical analysis of the event-related data, null trials (silent events) were randomly presented between the auditory stimuli. Participants were instructed to maintain their practice during the presentation of the sounds. During the meditation and neutral states, eyes remained open and directed toward a fixation point on a black screen. In this study we did not include a behavioral task because practitioners reported that a task would disrupt their ongoing meditation.

We did, however, collect self report information about the quality of the blocks of meditation from all participants. After each scan run, participants were asked to verbally report the meditative intensity of each block on a scale from 1 to 9. Some participants, not comfortable using the number scale to quantify or qualify their meditative states, simply identified the two blocks, from among the four recorded, that were the best and the worst of the day. Using these quantitative and/ or qualitative reports we chose only those blocks rated as either best and worst, or the two blocks from among the four having the highest and lowest ratings on the 9 point scale for inclusion in our analyses of good vs. poor blocks of meditation. Two scans were run on two separate days (1 day apart for experts, in general less than 1 week apart for novices) due to the length of the scan run. Standard data collection and analysis processing procedures were followed and are described in SI Methods.

### Data collection

MR images were collected with a GE Signa 3.0 Tesla scanner equipped with a high-speed, whole-body gradient and a whole-head transmit-receive quadrature birdcage headcoil. Whole-brain anatomical images were acquired at the end of each session using an axial 3D T1-weighted inversion-recovery fast gradient echo (or IR-prepped fast gradient echo) sequence. The field of view (FOV) was 240×240 mm with a 256×256 matrix. The slice thickness was 1–1.2 mm, with 0.9 by 0.9 mm in-plane dimensions. Functional data were collected using whole-brain EPI (TR = 2000, TE = 30 ms). For functional images, sagittal acquisition was used to obtain 30 interleaved 4 mm slices with a gap of 1 mm between slices. The resulting voxel size was 3.75 by 3.75 by 5 mm (FOV = 240 mm, matrix = 64×64).

To ensure a high signal-to-noise ratio in areas prone to susceptibility artifacts, the field inhomogeneities were lessened during data collection using high-order shim coils that applied small correction gradients. In addition, acquisition of a 3D field map of the magnetic field provided a complementary strategy to further reduce distortion (these data were not acquired for the first three experts). Based on these field maps, echo planar imaging (EPI) data were unwarped so that accurate alignment to anatomical images could be made [25], [26]. During the fMRI session, head movement was restricted using a vacuum pillow (Vac Fix System, S&S Par Scientific). A Silent Vision system (Avotec, Inc., Jensen Beach, FL) displayed the fixation point for the concentrative task. Eye movements, fixations and pupil diameter were continuously recorded during the fMRI scan using an iView system (sampling rate, 60 Hz) with a remote eye-tracking device (SensoMotoric Instruments, 2001). We collected pupil data from 13 controls and 10 experts participants.

### Data analysis

Analysis techniques were similar to those described previously by our lab [27]. Briefly, data processing was implemented via AFNI (Analysis of Functional Neural Images) version 2.51 software [28]. Data processing steps included image reconstruction in conjunction with smoothing in Fourier space via a Fermi filter, correction for differences in slice-timing, 6-parameter rigid-body motion correction, and removal of skull and ghost artifacts. The motion estimates over the course of the scan for translation (inferior-superior, right-left, and anterior-posterior) and rotation (yaw, pitch, roll) were charted. Time points with more than 0.5 mm of motion, as well as time points in which head motion correlated with the presentation of the block (which could lead to spurious activations that might be mistaken for brain activation) were removed from the analysis. Due to excessive head motion, 1 expert and 1 novice were omitted from the group analysis. Two of the experts could not complete the second session, and one of the two sessions was omitted for 5 of the experts and 6 of the novices, due to excessive head motion. One of the sessions of 1 novice was omitted due to sleepiness. Between the two subject groups, participants with only one session of data were matched (7 experts and 7 novices).

The time series of meditative blocks and neutral blocks were modeled with a least-squares general linear model (GLM) fit that modeled the block effect, event-related sound responses and motion parameters in six directions. For the event-related sound responses, a 6-parameter sine function basis set was used to model the shape of the hemodynamic response in a 20 second window. The average of the estimated event-related response between 2 seconds and 12 seconds was converted to percentage signal change using the mean overall baseline and spatially smoothed using a 6 mm Gaussian filter. The resultant percentage signal change maps were transformed into the standardized Talairach space via identification of anatomical landmarks on the high-resolution anatomical image.

The main analysis of the emotional sounds (event-related design) was performed using a 2×2×3 factorial design (voxelwise 3-way ANOVA) with State (resting and meditation states) and Valence (negative, neutral and positive) as factors varying within subject and with Group as a between-subjects factor (Matlab package for AFNI, C. Gang). Monte Carlo simulations were run to correct for multiple testing to achieve an overall corrected mapwise p = 0.05. For the State effect, Group by State effect and State by Valence interaction and Group by State by Valence, we found that the minimum cluster sizes were, respectively, of 323, 1030, 1580 and 3580 contiguous voxels with the data thresholded at an uncorrected voxelwise p-value of p = 0.001, p = 0.01, p = 0.02 and p = 0.05 respectively. The data were then overlaid onto a high-resolution anatomical image. Complementary analyses were then run on the average percentage signal change in each of these clusters (Tables 1–5) using the same factorial analysis. Second, we tested for hemispheric differences in level of activation. In order to create a symmetrical cluster common in size in both hemispheres, the larger cluster from one side of the brain was flipped to the other side, combined with the smaller one and flipped back to initial side. Finally, in the table describing the main effect for state (Table 3) we ran paired t-tests comparing responses during meditation and resting states within each group in each of these ROIs (paired two-tailed ttest, threshold p = 0.05).An a priori anatomical template was then used to further delineate overlapping areas. We chose to delineate the posterior vs. anterior temporal lobes at y = .−25 mm. Paired ANOVAs were run on each ROI with laterality (right and left clusters) and group (experts versus novices) as factors. A complementary analysis of the emotional sounds was performed using only the positive and negative sounds (2×2×2 factorial design, minimum cluster size 3580 voxels for the Group by State by Valence interaction at an uncorrected voxelwise p-value of p = 0.05). A exploratory voxel-wise analysis of the relationship between verbal report and BOLD signal during meditation was performed on average response to positive and negative emotional sounds using 2×2 factorial design with verbal report (poor vs. good blocks of meditation as verbally reported) as a factor varying within subject and with group as a between-subjects factor (Matlab package for AFNI, C. Gang). We found that the minimum cluster sizes were 1030 contiguous voxels for the main effect of verbal report and 1580 contiguous voxels for the Group X Verbal report interaction, with the data thresholded at an uncorrected voxelwise p-value of p = 0.01 and p = 0.02, respectively. Only 12 experts and 10 novices had sufficient verbally reported information to be included in this analysis. Finally, a regression was applied to only the experts to examine any effect the number of lifetime hours of training and age had on the percentage of signal changes in these clusters. Neither of these factors had any significant effect.

### Pupil diameter

Analysis was similar to Urry et al. (2006): the pupil dilation data were cleaned and processed using algorithms designed by Siegle, Granholm, and Steinhauer (2002, unpublished Matlab code) with Matlab software (MathWorks, Natick, MA) and adapted in our laboratory (L. L. Greischar, 2003, unpublished Matlab code). Blinks were identified and eliminated using local regression slopes and amplitude thresholds. Missing data points were then estimated using linear interpolation across artifacts shorter than 4 seconds in duration. Pupil diameter was aggregated into 1 s bins, and autonormalized compared to the mean and global variance across the session. The pupil dilation responses following the emotional sounds were normalized across participants by substracting the ongoing 1-second baseline preceding the stimulation. Irrelevant drifts in the pupil diameter data over the course of the scan session were removed by automatically rejecting trials that did not show the average phasic response to sounds. The group analysis was performed on the mean pupil diameter across the first 5 seconds following the end of the sound stimulus. Participants needed at least 6 trials in both the resting and meditation condition to be part of the group analysis (9 controls and 7 experts participants matched the above criteria). An analysis of variance was first conducted on the pupil data only (ANOVA, with State (resting and meditation states) as factor varying within subject and with Group as a between-subjects factor). An analysis of covariance was then conducted between the pupil data and the BOLD responses to sounds in the clusters showing a State or State by Group interactions (9 controls and 7 experts participants, ANCOVA with the pupil diameter as a continuous factor and State (resting and meditation states) as factor varying within subject. Insufficient power precluded treating Group as a factor).
